# The Development of a Viral Mediated CRISPR/Cas9 System with Doxycycline Dependent gRNA Expression for Inducible *In vitro* and *In vivo* Genome Editing

**DOI:** 10.3389/fnmol.2016.00070

**Published:** 2016-08-18

**Authors:** Christopher A. de Solis, Anthony Ho, Roopashri Holehonnur, Jonathan E. Ploski

**Affiliations:** School of Behavioral and Brain Sciences, Department of Molecular and Cell Biology, University of Texas at DallasRichardson, TX, USA

**Keywords:** CRISPR/Cas9, TRE3G, TET2, amygdala, AAV vectors, doxycycline, inducible, genome editing

## Abstract

The RNA-guided Cas9 nuclease, from the type II prokaryotic Clustered Regularly Interspersed Short Palindromic Repeats (CRISPR) adaptive immune system, has been adapted and utilized by scientists to edit the genomes of eukaryotic cells. Here, we report the development of a viral mediated CRISPR/Cas9 system that can be rendered inducible utilizing doxycycline (Dox) and can be delivered to cells *in vitro* and *in vivo* utilizing adeno-associated virus (AAV). Specifically, we developed an inducible gRNA (gRNAi) AAV vector that is designed to express the gRNA from a H1/TO promoter. This AAV vector is also designed to express the Tet repressor (TetR) to regulate the expression of the gRNAi in a Dox dependent manner. We show that H1/TO promoters of varying length and a U6/TO promoter can edit DNA with similar efficiency *in vitro*, in a Dox dependent manner. We also demonstrate that our inducible gRNAi vector can be used to edit the genomes of neurons *in vivo* within the mouse brain in a Dox dependent manner. Genome editing can be induced *in vivo* with this system by supplying animals Dox containing food for as little as 1 day. This system might be cross compatible with many existing *S. pyogenes* Cas9 systems (i.e., Cas9 mouse, CRISPRi, etc.), and therefore it likely can be used to render these systems inducible as well.

## Introduction

The CRISPR/Cas9 based genome editing system has proven to be an extremely powerful tool for scientists seeking to genetically manipulate cells and tissues *in vitro* and *in vivo* across multiple species. This genome editing system takes advantage of the RNA-guided Cas9 nuclease from the type II prokaryotic Clustered Regularly Interspersed Short Palindromic Repeats (CRISPR) adaptive immune system, where it has been adapted for the use of knocking out genes, creating specific modifications to genes and for the activation and suppression of transcription in a gene specific manner (Jinek et al., 2012; Cong et al., 2013; Gilbert et al., 2013; Larson et al., 2013; Mali et al., 2013; Qi et al., 2013; Sander and Joung, 2014; Tanenbaum et al., 2014). CRISPR/Cas9 can be used to genetically manipulate embryonic stem cells quickly and relatively easily, which has enabled genetically modified mice to be created significantly faster than conventional methodologies and this is especially true in cases where multiple genes are modified (Wang et al., 2013). This system also offers a potentially better alternative to RNAi mediated gene knockdown which requires the constant overexpression of an shRNA to mediate gene knockdown and in some cases has proven to be toxic when utilized *in vivo* (Grimm et al., 2006; McBride et al., 2008; de Solis et al., 2015).

The success of the CRISPR/Cas9 system can be explained, in part, by its simplicity. For example, this genome editing system can be reconstituted in eukaryotic cells simply by the presence of the Cas9 protein and guide RNA (gRNA) consisting of the fusion of a CRISPR RNA (crRNAs) and a fixed transactivating CRISPR RNA (tracrRNA)—just two genes are required. The first 20 nucleotides of the gRNA are custom designed to be complementary to the intended target site within the genome and consequently guide the Cas9 protein to this site, allowing Cas9 to create double strand breaks (DSB) of the targeted DNA. The DSB initiates the error prone nonhomologous end joining (NHEJ) DNA repair mechanism. Due to the error prone nature of this repair pathway, insertions and deletions (Indels) can be created at the DSB break/repair site. If the DSB occurs within the protein coding region of a gene, a loss of protein function can occur due to: the deletion of relevant codons, an insertion of inappropriate codons, or the creation of indels that lead to a shift in the reading frame—collectively, leading to a null allele/gene knockout. Alternatively, if a donor DNA template is provided, Homology Directed Repair (HDR) can occur instead of NHEJ. This phenomenon can be harnessed to create precise modifications of the genome at specific loci (Cong et al., 2013; Mali et al., 2013; Wang et al., 2013).

Recently, the CRISPR/Cas9 system has been rendered amenable for delivery to cells *in vivo* via adeno-associated virus (AAV) (Ran et al., 2015; Swiech et al., 2015). Delivery of CRISPR/Cas9 via AAV could be useful for potential human gene therapy approaches that might intend to utilize CRISPR/Cas9 technology and it is also highly relevant for preclinical studies that could benefit from the delivery of CRISPR/Cas9 to tissues of living mammals. The field of behavioral neuroscience can greatly benefit from viral mediated genome editing because it can be used to knock out genes in discrete locations of the animal brain in a cell type specific manner to allow the interrogation of how a gene influences behavior and circuit function. However, in complex behavioral experiments it can be advantageous to induce the desired genetic manipulation at a specific time point during an ongoing experiment, but we are limited in our ability to temporally control when genome editing will occur utilizing existing technology.

Utilizing CRISPR/Cas9 systems *in vivo*, in mammals, may allow these systems to be active for days to weeks at a time. In cases where CRISPR/Cas9 might be virally delivered to humans for gene therapy, the CRISPR/Cas9 system could be active indefinitely. In these instances, where CRISPR/Cas9 is chronically active for extended durations, there might be an increase in the accumulation of off-target editing/mutations. Thus, for *in vivo* applications, it would likely be beneficial to minimize the duration of Cas9 mediated genome editing to the time necessary for Cas9 to edit its intended target site and this has been, in part, the motivation for developing previously described inducible CRISPR/Cas9 genome editing systems (Davis et al., 2015; Nihongaki et al., 2015; Zetsche et al., 2015). However, while some of CRISPR/Cas9 systems have been adapted for AAV delivery (Ran et al., 2015; Swiech et al., 2015; Karnan et al., 2016; Yang et al., 2016), none of these AAV based systems, to date, have been adapted to allow the genome editing function to be temporally regulated.

Due to the benefits of possessing temporal control over the duration of Cas9 mediated genome editing, we sought to develop an inducible CRISPR/Cas9 system that could be virally delivered and regulated utilizing Doxycycline (Dox). In our first set of experiments, we attempted to regulate Cas9 expression from a tetracycline response element containing promoter; however, we determined that genome editing could not be regulated in a Dox dependent manner, due to the leakiness of Cas9 expression within this system. As an alternative, we developed a viral vector that could regulate gRNA expression in a Dox dependent manner, and we demonstrate that our two vector CRISPR/Cas9 system can be virally delivered *in vivo* to the mouse brain and genome editing can be induced in a Dox dependent manner. The inducible gRNA vector (gRNAi) we developed is cross compatible with many existing *S. pyogenes* Cas9 (SpCas9) systems and therefore coupling our gRNAi AAV vector with these systems may enable them to be inducible as well (i.e., Cas9 mouse, CRISPRi, etc.).

## Materials and methods

### Plasmid construction

All viral plasmids generated for this study were produced utilizing standard recombinant DNA cloning techniques. The pAAV-pMecp2-SpCas9-spA plasmid (pX551) was a gift from Feng Zhang's laboratory (Swiech et al., 2015; Addgene #60957) and this vector served as the backbone for the inducible Cas9 vectors described in this study. The truncated P_Tight_ sequence was PCR amplified from pTRE-TIGHT-Cx43-eYFP [a gift from Robin Shaw (Smyth et al., 2010; Addgene plasmid # 31807)] and cloned into the AgeI-XbaI sites of pX551 to create the P_Tight_-Cas9 vector. This truncated P_Tight_ sequence contained 3 Tet operators. This vector was subsequently modified by cloning into the BsmI-EcoR1 sites a new C-terminus region of Cas9 that lacked the C-term NLS and instead contained the following core PEST degron sequence (HGFPPAVAAQDDGTLPMSCAQESGMDRH; Li et al., 1998) utilizing an appropriately designed gBlock (Integrated DNA technologies) to create the P_Tight_-Cas9PEST vector. This core PEST sequence is utilized in the destabilized eGFP variant, D1eGFP. To create the P_TRE3G_-Cas9PEST vector, the truncated P_TRE3G_ sequence was PCR amplified from pLenti CMVTRE3G eGFP Neo (w821-1; A gift from Eric Campeau) and cloned into the AgeI-KpnI sites of the P_Tight_-Cas9PEST vector. The truncated TRE3G promoter contains 2 Tet operators. The P_TRE3G_-Cas9PEST vector was subsequently modified by cloning in a new 5′ UTR/N-terminus region into the AgeI-BstXI sites, utilizing an appropriately designed gBlock (Integrated DNA Technologies), that lacked a Kozak sequence and the N-Terminal NLS to create the P_TRE3G_-ΔCas9-PEST viral vector. To create the gRNA/rtTA-GFP viral vector, the previously described AAV2 genome vector, pAAV-shRNA expression cassette vector, (Hommel et al., 2003) was systematically gutted and the appropriate sequences were subsequently cloned into it. First, the CMV-rtTA-GFP-Blastocidin S Resistance-WPRE expression cassette was removed from pMA2640 (Addgene, #25434; Alexeyev et al., 2010), and cloned into the XhoI-ClaI sites of the pAAV-shRNA vector. A short ~70 base pair 3′UTR sequence containing two polyadenylation signal sequences, which we've used previously (Holehonnur et al., 2015), was cloned into the ClaI-MluI sites. The Blastocidin S Resistance coding region was removed and the appropriate portion of GFP with a stop codon was cloned back into the BamHI-MroI sites. The gRNA expression cassette was PCR amplified from pX330 [Gift from Feng Zhang (Cong et al., 2013; Addgene, #42230)] using the following DNA primers, (gRNA RsrII FP ataCGGTCCGgagggcctatttcccatgattccttc, gRNA XhoI RP aacCTCGAGgccatttgtctgcagaattggcgcacg), and cloned into the RsrII-XhoI sites, to create the final AAV2-gRNA/rtTA-GFP viral vector. Because the BbsI sites used for cloning the gRNAs into the gRNA expression cassette were not unique to this AAV plasmid, the gRNAs were first cloned into the BbsI sites of the pX330 plasmid and then the entire gRNA expression cassette was transferred into the AAV plasmid via the RsrII-XhoI sites utilizing the above mentioned DNA primers. The DNA oligonucleotides coding for the Tet2 gRNAs compatible with the pX330 plasmid were previously described (Wang et al., 2013; Top: CACCGAAAGTGCCAACAGATATCC, Bot: AAACGGATATCTGTTGGCACTTTC). The AAV2-gRNAi-TetR viral vector was developed starting with the AAV:ITR-U6-sgRNA(backbone)-hSyn-Cre-2A-EGFP-KASH-WPRE-shortPA-ITR plasmid (Addgene, #60231; Platt et al., 2014) as a backbone. The CMV promoter driving the TetR coding region was PCR amplified from pQCXIN-TetR-mCherry plasmid [a gift from Tom Misteli (Roukos et al., 2013; Addgene, # 59417)] and it was inserted into this vector via the XbaI-NheI sites. This resulted in the removal of the hSyn-Cre region. Next, an inducible gRNA expression cassette containing an HI/TO promoter was created using an appropriately designed gBlock (Integrated DNA Technologies) and it was cloned into the MluI-Xbal sites, to create the AAV2-gRNAi-TetR viral vector. Guide RNA sequences can be cloned into this vector via the SapI sites, in a similar manner as to how gRNA sequences are cloned into the AAV:ITR-U6-sgRNA(backbone)-hSyn-Cre-2A-EGFP-KASH-WPRE-shortPA-ITR plasmid (Addgene, #60231; Platt et al., 2014) or the similar pX552, pAAV-U6sgRNA-hSyn-GFP-KASH-bGH vector (Addgene, # 60958). The Tet2 gRNA sequences used with this vector were (Top: ACCGAAAGTGCCAACAGATATCC, Bot: AACGGATATCTGTTGGCACTTTC). The entire HI/TO gRNAi expression cassette can be PCR amplified with the following DNA primers [H1TO gRNA FP (MluI) AGCTACGCGTAATATTTGCATGTC and H1TO gRNA RP (XbaI) ACGTTCTAGAACTAGTCCATGG]. The entire U6/TO and H1-L/TO expression cassettes containing the Tet2 gRNA were PCR amplified from appropriately designed gBlocks (Integrated DNA Technologies) and inserted into the AAV2-gRNAi-TetR viral vector via the MluI-XbaI sites. The DNA sequences for these new plasmids will be made available upon request. All gRNA viral plasmids will be made available through Addgene.

### Assessment of *in vitro* genome editing

For *in vitro* genome editing experiments, Neuro-2a cells (N2A; ATCC) were grown to a confluency of 60–65% in a 24 well cell culture plate and transfected with a plasmid containing a Cas9 transgene and a plasmid containing a gRNA transgene in a 1:1 ratio with Lipofectamine 2000 (Invitrogen), following the manufacturer's instructions. In cases where pX330_Tet2_ or pX330_Empty_ were used, only one plasmid was transfected. For samples that required Doxycycline (Dox), the media was replaced with fresh media 6 h post transfection that contained 10 μg/mL of Dox (Clontech). The cells were harvested 96 h post transfection and were collected by centrifugation (10 min @ 14,000 RPM). Genomic DNA was extracted using a the QIAamp DNA Mini Kit, (Qiagen, Cat #51304) following the manufacturer's instructions. A 466 base pair region containing the Tet2 gRNA target site was PCR amplified from genomic DNA in a standard 25 μl PCR reaction (Platinum Taq; Invitrogen) using the following previously described DNA primers (Tet2 Surv FP CAGATGCTTAGGCCAATCAAG and Tet2 Surv RP AGAAGCAACACACATGAAGATG; Wang et al., 2013). One hundred and fifty nanograms of isolated genomic DNA (described above) was used as the DNA template, and the PCR was performed with the following cycling parameters [94°C, 2 min; (94°C, 30 s; 54°C, 30 s; 72°C, 30 s) × 37 cycles, 72°C, 45 s]. Amplification was confirmed using gel electrophoresis on a 1.5% agarose, 1 X TAE gel. Three microliters of PCR product was digested with 5 units of EcoRV-HF (New England Biolabs) for 2 h in a standard 10 μl restriction enzyme reaction following the manufacturer's instructions. Each 3 μL of digested PCR product was electrophoresed on a 2.0% agarose, 0.5 X TBE gel to determine if genome editing had occurred. In some cases, the amount of genome editing was quantified using ImageJ (U.S. National Institutes of Health, Bethesda, Maryland, USA, http://imagej.nih.gov/ij/). This was accomplished by measuring the optical density peaks using the gel analysis feature. The peaks for the cut DNA and non-cut DNA were selected and the label peaks feature was used to determine the percentage of cut and non-cut DNA in each individual sample. Some *in vitro* samples were screened using the resolvase based mutation detection kit (Guide-it Mutation Detection Kit, Clontech, Cat #631443) following the manufacturer's instructions.

### Immunocytochemisty (ICC)

Glass coverslips were placed in 24 well cell culture plates and treated with poly-l-lysine (0.1 mg/mL; Sigma) overnight. The following day, the poly-l-lysine was removed and the coverslips were washed 3 × with phosphate buffered saline pH 7.4 (PBS). N2A cells were seeded at 65% confluency on these glass coverslips. Twenty-four hours later, the cells were transfected with AAV viral plasmids designed to express Cas9 and rtTA/gRNA plasmids using Lipofetamine 2000 (Invitrogen), following the manufacturer's instructions. Six hours post transfection, media was replaced with fresh media and in some cases the media contained 10 μg/mL of Dox. The ICC procedure was carried out 24 h post addition of doxycycline as previously described (Holehonnur et al., 2015) using an anti-Cas9 antibody (1:200; Diagenode, C15200203) and TxRed secondary antibody (1:000; Life Technologies). The ICCs were imaged at 200X magnification using a fluorescence microscope (Olympus, BX51). The on and off Dox pictures were taken at the same exposure conditions. An additional high exposure image was taken of the off Dox samples to reveal the low level Cas9 expression. For ICCs following viral transduction of 293FT cells, 1.6 μl of AAV2/DJ-P_Tight_-Cas9 (6E12 GC/mL) and 1 μl of AAV2/DJ-gRNA/rtTA-GFP (1E13 GC/mL) were diluted in 200 μl of culture media containing 2% FBS and it was applied to cells for 2 h, with gentle rocking every 30 min. Following the 2-h incubation, 400 μl of culture media containing 18% FBS was added to the wells. Dox was added to the wells 6 h later and the ICC was carried out 24 h after the addition of Dox.

### Viral production, purification, and titering

Procedures were carried out as previously described (Holehonnur et al., 2014). AAV2 genome plasmids were pseudotyped as either AAV2/DJ8 or AAV2/DJ as specified. Viruses were produced using a triple-transfection, helper-free method into 293FT cells (Invitrogen) using polyjet (Signagen Laboratories) following the manufacturer's instructions for AAV production. A total of 5 × 15 cm cell culture plates were transfected per virus. Viruses were purified on an iodixanol step gradient and further concentrated and purified using Amicon Ultra-15 centrifugal filter units (Millipore). Purified AAV was titered using a quantitative-PCR based titering method as previously described (Holehonnur et al., 2014). All Cas9 transgene containing viruses were titered utilizing custom Cas9 primers/probe (Custom Taqman gene expression assays, Invitrogen). All GFP transgene containing viruses were titered with (GFP Primer/Probe (ID# Mr04329676_mr; Invitrogen). The results were reported as the number of DNAse resistant viral particles as genome copies per milliliter (GC/ml).

### Viral infusion

Viral infusions targeting the mouse BLA were performed similarly as described (Holehonnur et al., 2014). Briefly, mice were rendered unconscious with an intra-peritoneal injection of ketamine (100 mg/kg) and xylazine (10 mg/kg) prior to stereotaxic surgery. Thirty-one gauge custom infusion cannulas (C315G, PlasticsOne) were very securely inserted into polyethylene tubing (I.D. 0.0150 in. O.D. 0.043 in., wall thickness 0.0140 in; A-M systems, Inc.) that were 50 cm in length. These tubes were first backfilled with 1 x phosphate buffered saline, pH 7.4 (PBS), followed by sesame oil, where only 1 × PBS was present in the ~5 cm region closest to the infusers to avoid getting sesame oil in the brain. Syringes (2 μL, 23-gauge (88,500); Hamilton Company)) were used for the viral infusion. The viral cocktail was drawn up into the infusion cannula. Infusers were bilaterally lowered into the BLA of mice [AP +1.6, ML ± 3.3, −DV ± 4.97] and infused (1 μL/side) at a rate of 0.07 μL/min for 15 min using an infusion pump (New Era Pump Systems Inc., NE-300) The AAV2/DJ-P_Tight_-Cas9 and AAV2/DJ-gRNA_Tet2/Empty_/rtTA-GFP viruses were co-infused bilaterally into the BLA, each at a titer of 6E12 GC/mL (1 μl/side). The AAV2/DJ8-P_Mecp2_-Cas9 and AAV2/DJ8-gRNAi_Tet2/Empty_ viruses were co-infused bilaterally into the BLA, each at a titer of 2.5E12 GC/mL (1 μl/side). In cases when mice were provided Dox, it was supplied through their food (Dox 1 g/kg; Bio-Serv).

### Assessment of *in vivo* genome editing

At the appropriate time point, mice were euthanized via CO_2_ euthanasia and the brain was rapidly removed and frozen with powdered dry ice and then stored at −80°C. Tissue was then mounted and sliced using a cryostat (Thermo Scientific) to take coronal sections that contained the amygdala. When GFP reporter signal was present in the amygdala, 200 μm punches were taken within the amygdala with a 1 mm punch tool (Fine Science Tools) until the GFP signal ceased within the amygdala. Punched tissue was stored at −80°C. Tissue was homogenized in a 1.7 mL centrifuge tube with pestle and extracted in the same manner as the *in vitro* experiments.

### Sequencing of edited DNA

N2A cells that were transfected with pX330_Tet2_ were prepared for DNA extraction and PCR amplification as described above. PCR product was digested with EcoRV and electrophoresed and the DNA band exhibiting editing was excised from the gel and purified using a gel purification kit (Qiagen). Gel purified DNA was cloned into the PCR4 Topo vector using a Topo cloning kit (Invitrogen) and transformed into Top10 cells (Invitrogen). Colonies were screened for the presence of the Tet2 sequence via PCR and PCR product was sent for DNA sequencing (Retrogen Inc.).

### Subjects

Adult C57BL/6 mice were used in this study. All animals were housed individually and maintained on a 12 h light/dark cycle. Food and water were provided *ad libitum* throughout the experiments. Animal use procedures were in accordance with the National Institutes of Health Guide for the Care and Use of Laboratory Animals and were approved by the University of Texas at Dallas Animal Care and Use Committee.

### Statistical analysis

Quantified genome editing data was analyzed using a non-parametric Kruskal–Wallis individual sample comparison or multiple sample comparison, with a probability threshold of 0.05. A one-way ANOVA, followed by a Fisher's PSD *post-hoc* analysis was used to compare the different inducible promoters. A two-tailed *T*-test assuming equal variances was used to compare samples analyzed with the mutation detection kit.

## Results

### Construction of a doxycycline regulated SpCas9 transgene within an AAV2 viral vector

**Figure 1:**
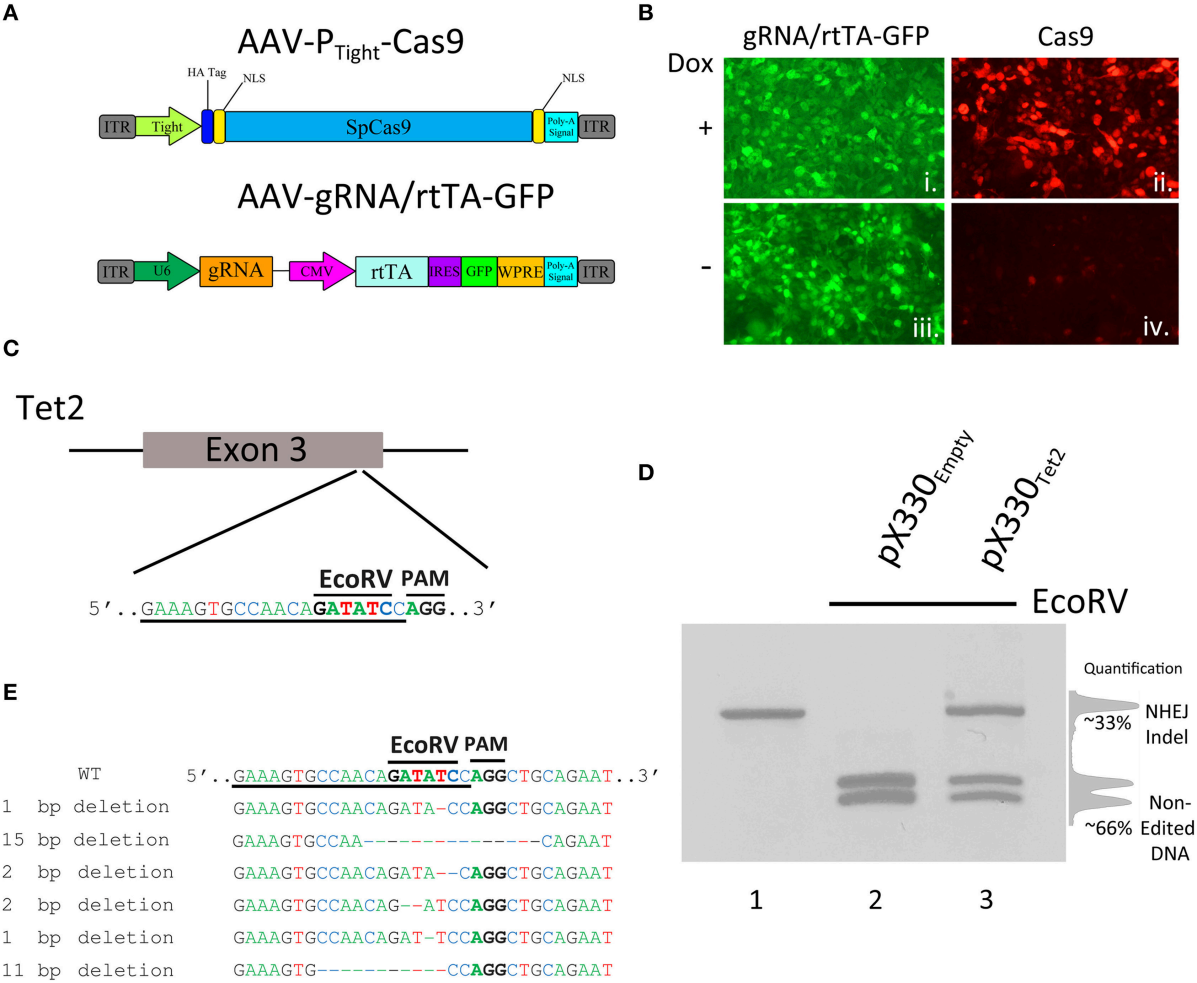
**(A)** AAV vector maps depicting AAV-P_Tight_-Cas9 and AAV-gRNA/rtTA. AAV-P_Tight_-Cas9 consists of a Cas9 transgene under the control of a Dox inducible Tight promoter. AAV-gRNA/rtTA consists of a gRNA expression cassette and a rtTA (Tet-On Advanced) transgene controlled by a CMV promoter. It also is designed to express GFP via an IRES element following the rtTA reading frame. **(B)** ICC for Cas9 and GFP was performed on 293FT cells transduced by AAV-P_Tight_-Cas9 and AAV-gRNA/rtTA viruses in the presence or absence of Dox. Native GFP expression is visible in virtually all of the cells (i, iii). Cas9 expression is robustly induced in the presence of Dox (ii), compared to the no Dox condition (iv). Representative images are shown. The experiment was repeated twice with similar results. **(C)** Diagram depicting the approximate location of where the Tet2 gRNA targets the Tet2 locus. Underlined nucleotides indicate the sequence of the Tet2 gRNA. Location of the EcoRV site and PAM sequence are denoted. **(D)** An approximate 460 bps region of the Tet2 locus that includes the site targeted for editing via the gRNA_Tet2_, was PCR amplified from N2A genomic DNA and electrophoresed on a standard agarose gel and stained with ethidium bromide (lane 1). N2A cells were transfected with the pX330_Empty_, a plasmid designed to express spCas9 and no gRNA, and 96 h later, the genomic DNA was isolated and the Tet2 locus was PCR amplified and subjected to EcoRV digestion. The PCR product was cut into two pieces of DNA as expected (lane 2). However, when N2A cells were transfected with pX330_Tet2_ and similarly processed, the PCR product was incompletely digested resulting in a total of three bands on the gel - one uncut PCR product (~460 bps) and two smaller bands. In this case the genome editing was ~33%. **(E)** Edited DNA depicted in (**D**, lane 3) was gel purified and TA cloned and 6 independent clones were sequenced. These 6 clones contained deletions which destroyed the EcoRV site.

Viral vectors are necessary to introduce the genetic components of the CRISPR/Cas9 system into specific mammalian cells *in vivo*. Adeno-associated virus (AAV) is the vector of choice to use within mammalian systems due to its high tolerability *in vivo* (Daya and Berns, 2008) and because it is possible to produce high titer AAV relatively easily that is capable of transducing a large number of cells. In our first set of experiments, we focused on designing an inducible CRISPR/Cas9 system that was suitable for AAV delivery, where the SpCas9 transgene expression could be regulated. To accomplish this, it was necessary to fit the entire Cas9 transgene within AAV's strict genome packaging limit which hovers somewhere between 4.7 and 5 kb. This packaging limit also includes the two inverted terminal repeats that are ~150 bases each, therefore limiting AAV's ability to deliver transgenes that are no larger than ~4.4–4.7 kb. Since the coding region of SpCas9 is ~4.2 kb, this leaves very little room for the promoter and 3′ untranslated sequences (UTR). Because of this, we opted to design an AAV vector that harbored the Cas9 coding region under the control of a truncated 2nd generation tetracycline response element (TRE) promoter, P_Tight_ (Figure 1A). Of the three generations of TRE promoters (TRE2, Tight, TRE3G), the Tight promoter is the shortest of the three promoters. We also truncated the promoter at the 5′ end so that it only included 3 Tet operators (TetO), creating a promoter that was ~175 bps. To regulate the P_Tight_-Cas9 transgene and deliver the gRNA expression cassette, another AAV was developed, that harbored a gRNA transgene, utilizing a U6 promoter, and an additional transgene, containing a CMV promoter driving rtTA (Tet-On Advanced) transcription factor expression. This virus was also designed to express green fluorescent protein (GFP) from an internal ribosomal entry site (IRES), following the rtTA open reading frame (ORF; Figure 1A). In cells that receive both the P_Tight_-Cas9 virus and the gRNA/rtTA virus in the presence of Doxycycline (Dox), rtTA would become activated, allowing it to bind to the P_Tight_ promoter and drive Cas9 expression, inducing genome editing. When Dox is not present, the system theoretically should not be able to express Cas9 and therefore genome editing should not be possible. We produced both of these viral vectors pseudotyped as DJ serotype and transduced 293FT cells grown in culture in the presence or absence of Dox. Forty-eight hours post viral transduction, immunocytochemistry (ICC) was performed for Cas9 protein expression. The cells exhibited GFP fluorescence, indicating that they were efficiently transduced by the gRNA/rtTA-GFP virus. The ICC revealed a dramatic increase in Cas9 expression in the presence of Dox, indicating that our viral vectors appropriately expressed their intended transgenes and it appeared that Dox could regulate Cas9 expression (replicates of samples produced similar results; Figure 1B).

Next, we wanted to determine if these viral vectors could edit the genome in an inducible manner. To do this we chose to utilize a previously described and validated gRNA targeting the mouse Tet2 genomic locus. Tet2 is a member of the Ten-eleven translocation (Tet) gene family, and is involved in the enzymatic conversion of 5-methylcytosine (5 mC) to 5-hydroxymethylcytosine (5 hmC) to promote DNA demethylation. In particular, this gRNA is designed to target a genomic sequence that contains an EcoRV restriction enzyme site that is directly adjacent to the Protospacer Adjacent Motif (PAM) sequence (Figure 1C). The PAM site is the DNA sequence immediately following the DNA sequence targeted by the Cas9 nuclease. The location of the restriction enzyme site provides the ability to screen cells for genome editing, because Cas9 mediated genome editing utilizing this gRNA would likely destroy the enzyme site, and therefore genome editing can be accessed via restriction fragment length polymorphism (RFLP) analysis to assess the presence or absence of this restriction enzyme site. To demonstrate this and to validate that this Tet2 targeting gRNA indeed was capable of efficiently editing its intended target site, we transfected the mouse neuroblastoma cell line Neuro-2A (N2A), with the Cas9/gRNA expression plasmid pX330, that contained the Tet2 gRNA (pX330_Tet2_). Ninety-six hours post transfection, the cells were harvested and genomic DNA was isolated, and the Tet2 locus was PCR amplified utilizing PCR primers that flank the intended Cas9 cut site (Figure 1D, lane 1). The full length uncut Tet2 PCR product is 466 bps. In cells that did not receive gRNAs targeting the Tet2 locus (pX330_Empty_), genome editing did not occur, and the EcoRV digestion of the Tet2 PCR product yielded 2 bands at ~200 bps as expected (Figure 1D, lane 2). However, in cells that were transfected with the pX330_Tet2_ plasmid, genome editing did occur, as demonstrated by the fact that the EcoRV digestion was not capable of digesting the entire pool of PCR product. In this case, the digestion of the PCR product yielded fully digested DNA, creating 2 bands at ~200 bps and one undigested band at ~440 bps, indicating genome editing had occurred. In this case, the amount of editing was ~33% as determined by the ratio of the digested and undigested bands (Figure 1D, lane 3). We cloned and sequenced some of these edited PCR products to better characterize the nature of Cas9 mediated Indel formation. In the 6 independent clones we sequenced, Cas9 had created deletions spanning 1–15 nucleotides, therefore underscoring the utility of the RFLP screening (Figure 1E).

### A doxycycline regulated SpCas9, within AAV, exhibits leaky expression, and genome editing in a doxycycline independent manner

**Figure 2:**
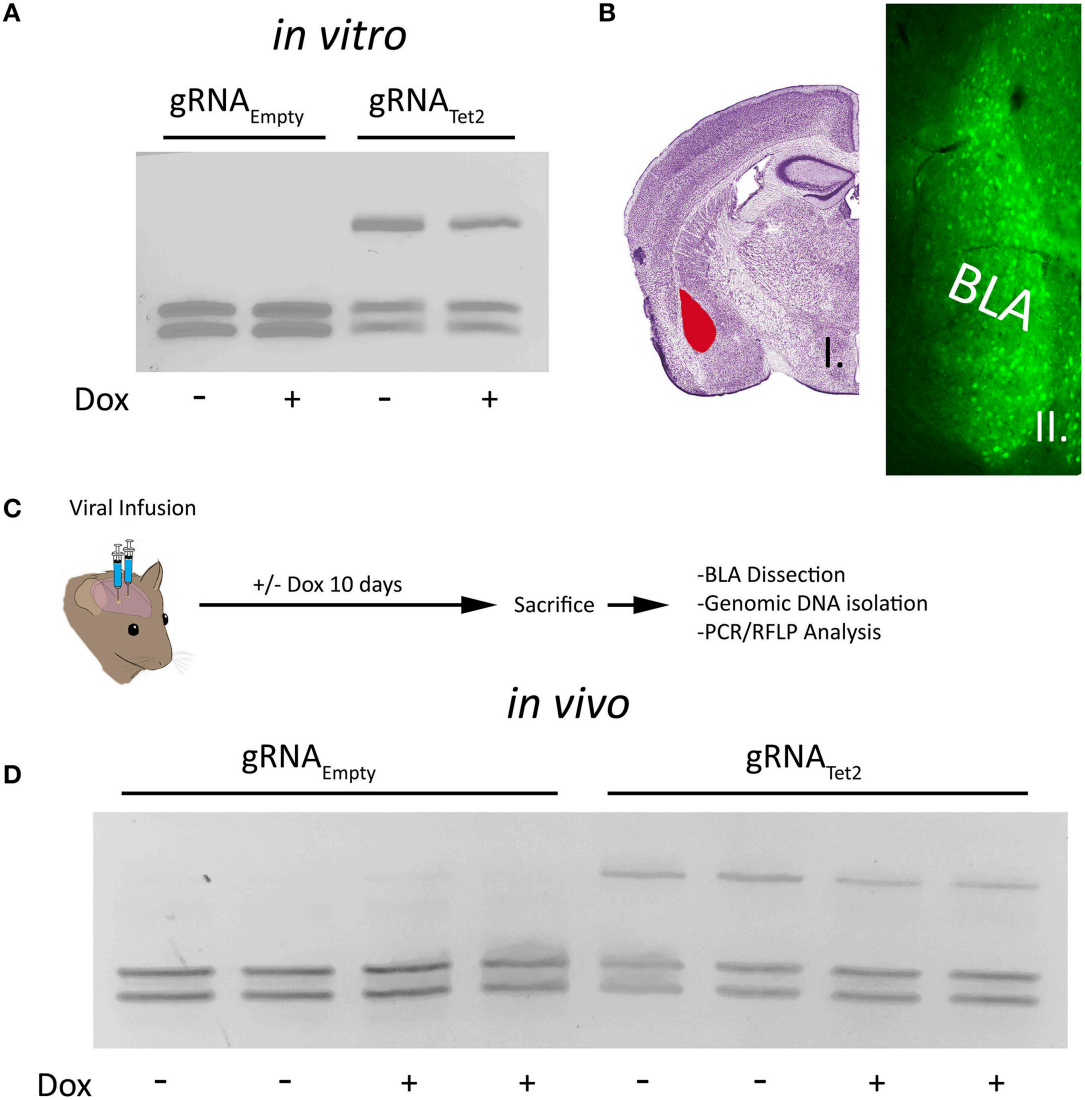
**(A)** The AAV-P_Tight_-Cas9 plasmid and the AAV-gRNA_Tet2_-rtTA plasmid were cotransfected into N2A cells in the presence or absence of Dox. As a control, the AAV-P_Tight_-Cas9 plasmid and the AAV-gRNA_Empty_-rtTA plasmid were co-transfected too. Nighty six hours post transfection, the cells were harvested, the genomic DNA was isolated and PCR and RFLP analysis was performed to assess if genome editing had occurred at the Tet2 locus. Genome editing was not observed in cells that had received the gRNA_Empty_ plasmid either in the presence or absence of Dox as expected. Cells that were transfected with a plasmid containing the gRNA_Tet2_ plasmid, did exhibit editing, however the editing occurred in samples that were in the presence or absence of Dox, indicating that genome editing occurred independently of Dox. Similar results were observed in at least 3 independent samples per group. **(B)** Image of a Nissl stained coronal mouse brain section with the BLA highlighted in red [adapted from (Franklin and Paxinos, 2007)] (i). Image depicting GFP expression within BLA neurons in a similar anatomical coronal slice as in (i), taken 10 days following BLA infusion of both AAV-gRNA_Tet2_-rtTA-GFP and AAV-P_Tight_-Cas9 viruses (ii). **(C)** Timeline from infusion of virus into the mouse amygdala to assessment of genome editing via RFLP analysis. **(D)** RFLP analysis of genome editing from BLA tissue transduced with AAV-P_Tight_-Cas9 and AAV-gRNA_Empty_-rtTA or AAV-P_Tight_-Cas9 and AAV-gRNA_Tet2_-rtTA from mice that were fed a diet that included Dox or a diet that did not include Dox. No genome editing occurred in samples that received gRNA_Empty_ as expected. Genome editing did occur in BLA samples that received gRNA_Tet2_ however, genome editing occurred independently of Dox administration. Two independent samples per group are shown. Similar results were observed in at least 3 independent samples per group.

To determine if our P_Tight_-Cas9 and gRNA/rtTA-GFP viral plasmids could edit the Tet2 locus in a Dox dependent manner, we cotransfected the P_Tight_-Cas9 plasmid with the gRNA_Tet2_/rtTA-GFP plasmid containing the Tet2 gRNA into N2A cells in the presence and absence of Dox. As a control, we also cotransfected the P_Tight_-Cas9 plasmid with the gRNA_Empty_/rtTA-GFP plasmid that did not contain a gRNA into N2A cells in the presence or absence of Dox. Ninety-six hours post transfection, the cells were harvested and the genomic DNA was isolated and examined for genome editing at the Tet2 locus via PCR/RFLP analysis as described above. Cells that received the P_Tight_-Cas9 and gRNA_Empty_/rtTA-GFP plasmids, did not exhibit editing of the Tet2 locus, as expected. Cells that received the P_Tight_-Cas9 and the gRNA_Tet2_/rtTA-GFP plasmids did exhibit editing of the Tet2 locus; however, the editing occurred in the presence and the absence of Dox (similar results obtained in 3 independent samples per group; Figure 2A). Notably, there was no difference between the induced (i.e., Dox) samples, compared to the non-induced samples (i.e., no Dox), indicating that there might be a low level of Cas9 expression that is sufficient for genome editing in the non-induced samples. To determine if these viruses would function in an inducible manner *in vivo*, we infused these viruses into the basal and lateral amygdala (BLA) of mice. Specifically, AAV2/DJ-P_Tight_-Cas9 and AAV2/DJ-gRNA_Tet2_/rtTA-GFP were co-infused bilaterally into the BLA, each at a titer of 6E12 GC/mL (1 μl/side) (Figure 2B). For a control, AAV2/DJ-P_Tight_-Cas9 and AAV2/DJ-gRNA_Empty_/rtTA-GFP were infused in a similar manner. Half of the animals in the Tet2 group and half of the animals in the Empty group were placed on a diet of 1 g/kg of Dox. Ten days following surgery, the animals were sacrificed, and their BLA were microdissected. The genomic DNA was isolated from the microdissected BLA tissue and subjected to PCR/RFLP analysis to assess genome editing at the Tet2 locus (Figure 2C). Animals that received the AAV2/DJ-P_Tight_-Cas9 and AAV2/DJ-gRNA_Empty_/rtTA-GFP viruses, did not exhibit editing of the Tet2 locus, as expected. Animals that received the AAV2/DJ-P_Tight_-Cas9 and AAV2/DJ-gRNA_Tet2_/rtTA-GFP viruses did exhibit editing at the Tet2 locus, but similarly to the *in vitro* experiments, the editing was not Dox dependent (similar results obtained in 3 independent samples per group; Figure 2D). Since these viral plasmids cannot be tightly regulated using Dox, these vectors would not be suitable for inducible genome editing *in vitro* or *in vivo*.

**Figure 3:**
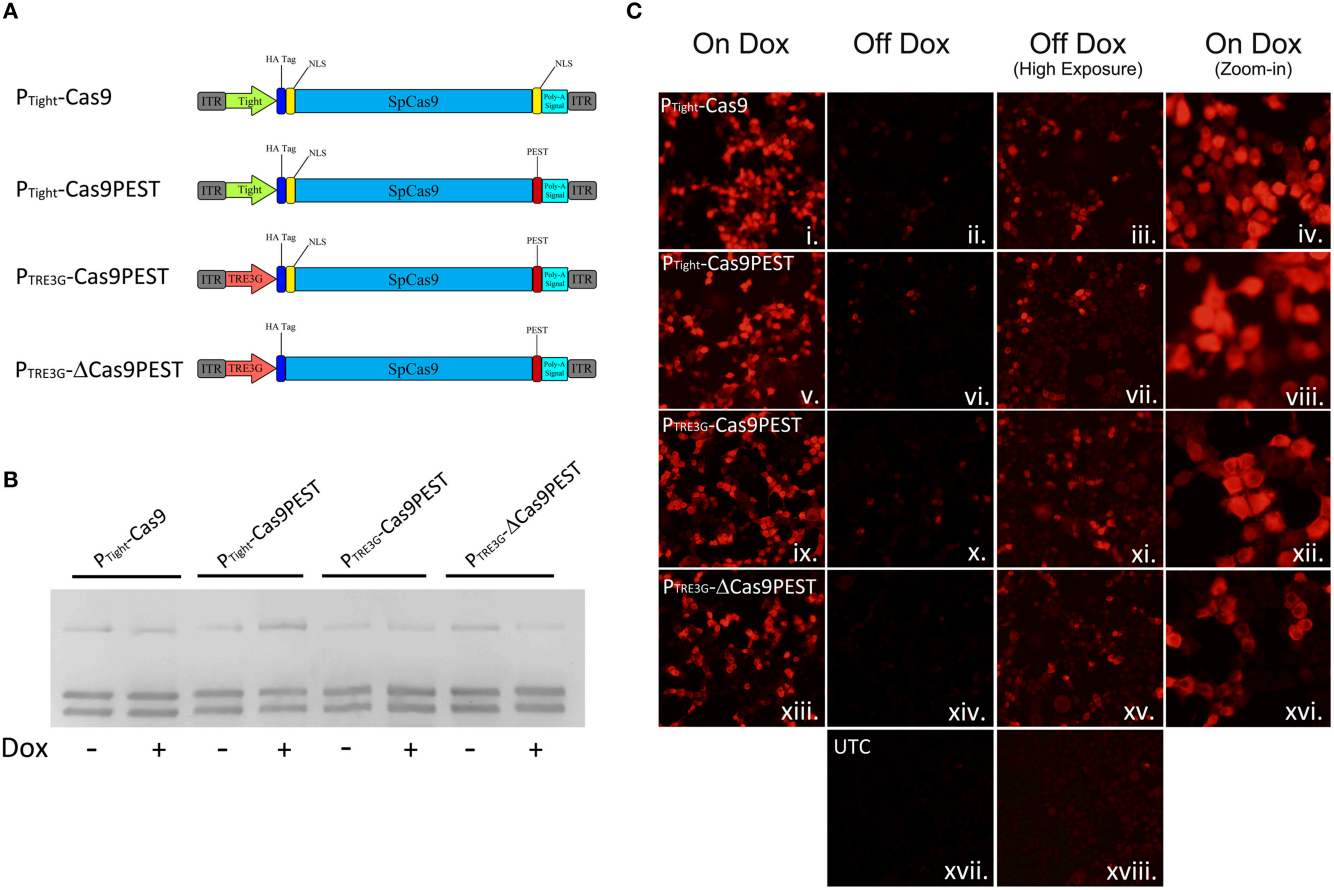
**(A)** AAV vector maps depicting AAV-P_Tight_-Cas9 and three new vectors derived from AAV-P_Tight_-Cas9. The C-terminal NLS was removed from AAV-P_Tight_-Cas9 and replaced with a PEST sequence to create AAV-P_Tight_-Cas9PEST. Then the Tight promoter was exchanged for a TRE3G promoter to create P_TRE3G_-Cas9PEST. Next, the kozak sequence and N-term NLS was removed to create P_TRE3G_-ΔCas9PEST. **(B)** The plasmids described in **(A)**, were co-transfected with AAV-gRNA_Tet2_-rtTA into N2A cells in the presence or absence of Dox. Nighty six hours post transfection, the cells were harvested, the genomic DNA was isolated and PCR and RFLP analysis was performed to assess if genome editing had occurred at the Tet2 locus. None of the Cas9 plasmids exhibited genome editing that was Dox dependent. Similar results were observed in at least 3 independent samples per group. **(C)** These Cas9 plasmids were transfected into N2A cells in the presence or absence of Dox and an ICC for Cas9 was performed. Images depict cellular Cas9 expression from cells treated with Dox (i, v, ix, and xiii) or off Dox (ii, vi, x, xiv, and xvii). These images were taken the same exposure. Images depicted in (iii, vii, xi, xv, and xviii) are of the off Dox samples at a higher exposure, which reveal low amounts of Cas9 expression in the absence of Dox. Images depicted in (iv, viii, xii, and xvi) are a zoom-in of the on Dox samples that shows a decreased nuclear localization of Cas9 protein for the P_TRE3G_-ΔCas9PEST group. Samples were processed in duplicate with similar results. UTC, untransfected control.

We speculated that the inability to regulate genome editing with our P_Tight_-Cas9 vector was due to low level Cas9 expression that was not rtTA dependent. Therefore, we created a number of other AAV vectors with modifications to attempt to reduce expression of Cas9 (Figure 3A). We first modified the P_Tight_-Cas9 vector by removing the C-terminal NLS sequence at the end of the Cas9 ORF and adding a PEST sequence (P_Tight_-Cas9PEST). The removal of the NLS should decrease the efficiency of Cas9 nuclear import which could reduce Cas9 activity and the addition of the PEST sequence could reduce the half-life of the Cas9 protein, thus reducing Cas9 levels. We subsequently modified P_Tight_-Cas9PEST by exchanging the Tight promoter for a truncated 3rd generation TRE3G promoter which contained 2 TetOs in hopes that the TRE3G promoter might lead to lower background Cas9 expression (P_TRE3G_-Cas9PEST). Finally, we modified the P_TRE3G_-Cas9PEST vector by removing the Kozak sequence and the N-terminal NLS (P_TRE3G_-ΔCas9PEST). The removal of the Kozak sequence, might lead to less efficient translation initiation, possibly lowering Cas9 protein levels, and the removal of the N-Terminal NLS should reduce the efficiency of Cas9 nuclear import, leading to reduced Cas9 activity. These 4 viral vectors were transfected into N2A cells with the gRNA_Tet2_/rtTA-GFP and gRNA_Empty_/rtTA-GFP vectors in the presence or absence of Dox and genome editing of the Tet2 locus was assessed via PCR/RFLP analysis as described above (Figure 3B). Genome editing of the Tet2 locus did not occur in the gRNA_Empty_ groups as expected (Data not shown). Cells that received the Cas9 viral plasmids and the gRNA_Tet2_/rtTA-GFP plasmid did exhibit editing of the Tet2 locus, however, there was virtually no differences in editing in the presence or the absence of Dox (similar results obtained in 3 independent samples per group). Next, we transfected N2A cells with the four Cas9 viral plasmids and the gRNA_Tet2_/rtTA-GFP viral plasmid in the presence or absence of Dox. Twenty-eight hours post transfection, the cells were examined by immunocytochemistry (ICC), to observe Cas9 expression (Figure 3C). In all cases there was a clear induction of Cas9 expression due to the presence of Dox. Using exposure conditions that were optimal for visualizing Cas9 expression in the presence of Dox, there appeared to be very little, if any, Cas9 expression in the absence of Dox. However, at a much higher exposure time there was obvious Cas9 expression in the absence of Dox, which explains the genome editing we observed in the absence of Dox (samples performed in duplicate with similar results). Interestingly, these findings underscore that very little Cas9 protein is needed for efficient genome editing. We did not observe obvious differences in Cas9 expression across these four Cas9 viral constructs, indicating that the presence of the PEST sequence, the use of the TRE3G promoter and removal of the Kozak sequence had little influence over Cas9 steady state protein levels in the on or off Dox conditions. The removal of the N and C terminal Cas9 NLSs did have a noticeable influence on the steady state Cas9 cellular localization, which became predominantly localized to the cytoplasm.

### AAV gRNAi vector exhibits doxycycline dependent genome editing *in vitro* and *in vivo*

**Figure 4:**
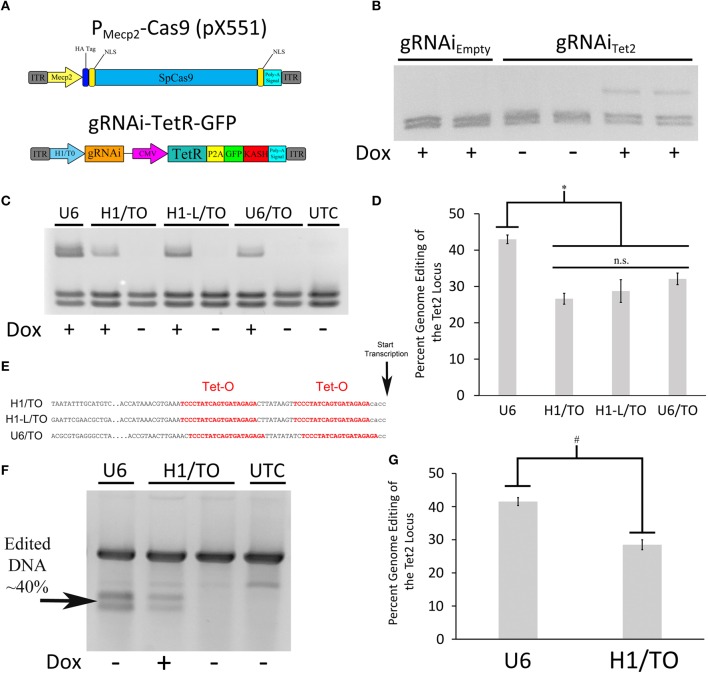
**(A)** AAV vector maps depicting the AAV-P_Mecp2_-Cas9 vector and an inducible gRNA vector, AAV-gRNAi-TetR-GFP. **(B)** The gRNAi plasmid described in **(A)**, containing either a Tet gRNA or no gRNA (Empty), were co-transfected with pX330_Empty_ into N2A cells in the presence or absence of Dox. Nighty six hours post transfection, the cells were harvested, the genomic DNA was isolated and PCR, and RFLP analysis was performed to assess if genome editing had occurred at the Tet2 locus. Genome editing was not observed in cells that had received the gRNA_Empty_ plasmid either in the presence or absence of Dox as expected. Cells that were transfected with a plasmid containing the gRNA_Tet2_ plasmid, did exhibit editing in the presence of Dox, but not in the absence of Dox, indicating that genome editing could be regulated in a Dox dependent manner. Similar results were observed in at least 3 independent samples per group. **(C)** The H1/TO promoter gRNA expression cassette was compared to gRNA expression cassettes composed of either a full length H1/TO promoter (H1-L/TO), a U6/TO promoter or a non-inducible U6 promoter (U6) for their ability to edit the Tet2 locus. Cells were transfected with the respective vectors in the presence or absence of Dox and genome editing was assessed 96 h post transfection (*n* = 4 per groups). **(D)** Quantification of genome editing revealed that the inducible promoter gRNA expression cassettes were not significantly different from each other (n.s. = *p* ≤ 0.0778) in their ability to edit the Tet2 locus and they were all slightly but significantly different from the non-inducible U6 promoter construct (^*^p ≤ 0.003). Four independent samples were screened with similar results. **(E)** Partial sequences of the inducible promoters used, highlighting the tetracycline operators (Tet-O) and transcriptional start site. **(F)** Genomic DNA from the same U6, H1/TO, and UTC samples used for **(C,D)** were subjected to a mutation detection kit (Clontech), in which the PCR amplified product was denatured, annealed and digested with Resolvase to directly detect edited DNA. **(G)** Levels of editing were comparable to those seen using the restriction digest PCR/RFLP analysis used for **(C,D)**. Again, levels of editing were significantly higher in the U6 group, compared to the H1/TO group (#*p* ≤ 0.005, Two Tailed *T*-test).

Since we were not able to generate an AAV based Cas9 expression system where Cas9 expression could be regulated using a TRE containing promoter, due to the leakiness of Cas9 expression within this system, we chose an alternative approach. We reasoned that if we could regulate gRNA expression, we could still generate an inducible viral mediated genome editing system. We generated a gRNA AAV vector containing a gRNA transgene controlled by a hybrid H1/TO promoter (gRNAi) similar to the promoter used in the BLOCK-iT inducible H1 RNAi vectors (Invitrogen; Figure 4A). This vector also contains a CMV promoter controlling the expression of TetR in frame with a self-cleaving P2A sequence followed by a GFP ORF fused to a KASH domain. Binding of TetR to the H1/TO promoter represses the gRNA transcription. The addition of Dox inhibits TetR binding and induces gRNA expression. The KASH domain will localize the GFP protein to the nuclear membrane, which will allow the nuclei of transduced neurons within the brain to be isolated away from non-transduced neurons using a combination of nuclei isolation via cellular fractionation and fluorescence-activated cell sorting (FACS). To test if the gRNA could regulate genome editing in a Dox dependent manner, we co-transfected the gRNAi_Tet2_-TetR viral vector containing a gRNA designed to edit the Tet2 locus with a Cas9 expression plasmid that did not contain a gRNA (pX330_Empty_) into N2A cells, in the presence or absence of Dox. As a control, we co-transfected the gRNAi_Empty_-TetR viral vector which did not contain a gRNA with pX330_Empty_ in the presence or absence of Dox. Ninety-six hours post transfection, the genome editing of the Tet2 locus was assessed via PCR/RFLP analysis as described above. Cells that received the gRNAi_Empty_-TetR vector did not exhibit genome editing as anticipated. Cells that received the gRNAi_Tet2_-TetR viral vector in the presence of Dox, exhibited genome editing, but in the absence of Dox, there was no editing (similar results obtained in 3 independent samples per group; Figure 4B). These data indicated that the gRNAi-TetR vector could provide a plausible system for inducible viral mediated genome editing.

To test the efficiency of the H1/TO gRNA expression cassette, we compared its ability to mediate genome editing in *vitro* to two different inducible promoter based gRNA expression cassettes and a standard U6 based gRNA expression cassette (U6; pX552). We designed a gRNA expression cassette that included the full length sequence for the H1 promoter (H1-L/TO) and a U6/TO promoter based gRNA expression cassette, as described in Henriksen et al. (2007) (Figure 4E). These different vectors, were cotransfected with pX330_Empty_ into N2A cells, in the presence or absence of Dox for 96 h. PCR/RFLP analysis revealed that the U6 group exhibited moderately better editing compared to the U6/TO, H1/TO and H1-L/TO groups [*F*_(3, 12)_ = 13.157, *p* = 0.0004] (*p* < 0.003; Figures 4C,D). We suspect that the presence of the Tet Operators, may slightly interfere with the efficiency of gRNA expression, however the inducible gRNA vectors do exhibit significant Dox dependent genome editing.

In order to determine if our restriction enzyme screening method to detect genome editing was comparable to other common methods that are designed to detect genome editing, we screened the same U6 and H1/TO samples from above utilizing the Resolvase based mutation detection kit (Clontech). The PCR products were denatured, annealed and digested with Resolvase, an enzyme that makes a DSB at the site of mismatched DNA. The screening revealed a significant difference between the U6 and H1/TO groups [*t*_(6)_ = 4.332, *p* = 0.0049], as expected. Additionally, similar levels of editing were detected utilizing this mutation detection method compared to the PCR/RFLP restriction enzyme analysis method (Figures 4F,G).

**Figure 5:**
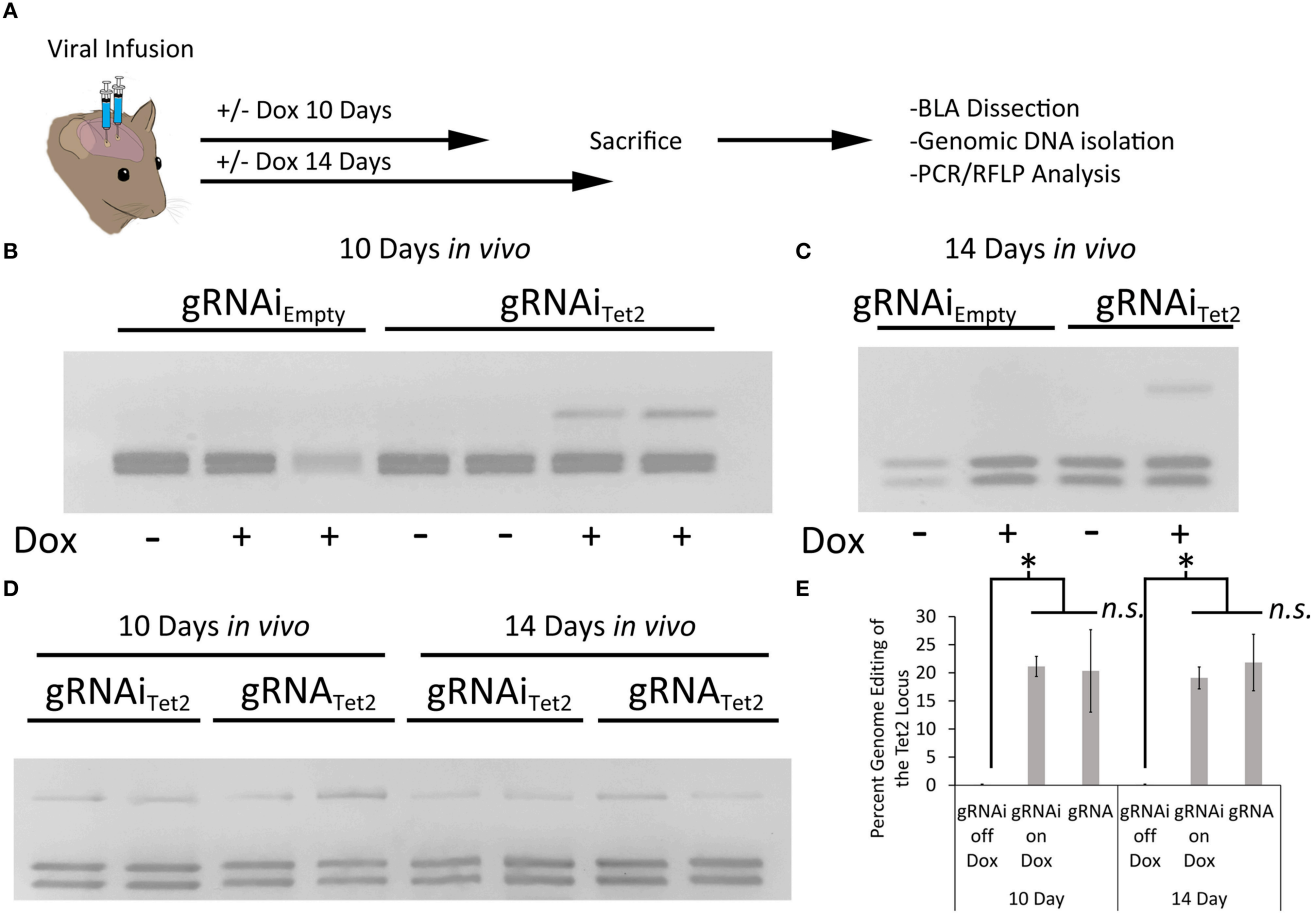
**(A)** Timeline from viral infusion to analysis of editing. Animals received either infusions of AAV-P_Mecp2_-Cas9 and AAV-gRNAi_Tet2_-TetR-GFP or AAV-P_Mecp2_-Cas9 and AAV-gRNAi_Empty_-TetR-GFP and were put on Dox or control (no Dox) food for 10 or 14 days post-infusion. Animals were then sacrificed and transduced cells within the amygdala were then microdissected and assessed for editing. **(B)** RFLP analysis of genome editing from BLA tissue transduced with AAV-P_Mecp2_-Cas9 and AAV-gRNAi_Tet2_-TetR-GFP or AAV-P_Mecp2_-Cas9 and AAV-gRNAi_Empty_-TetR-GFP from mice that were fed a diet that included Dox or a diet that did not include Dox and sacrificed 10 days post viral infusion. Genome editing did not occur in samples that received gRNA_Empty_ as expected. Genome editing did occur in BLA samples that received gRNA_Tet2_ and this genome editing was dependent on Dox administration. Two independent samples per group are shown. Similar results were observed in at least 3 independent samples per group. **(C)** RFLP Analysis at day 14 post-infusion showed comparable amounts of editing as compared to the day 10 post-infusion experiments described in **(B)**. One independent sample per group is shown. Similar results were observed in 3 independent samples per group. **(D)** A comparison of our inducible gRNAi virus was compared to a non-inducible system (gRNA) *in vivo*, following the same timeline for day 10 and 14 as described in **(B,C)**. **(E)** Quantification of total edited DNA in RFLP experiments revealed a similar degree of editing between the inducible and non-inducible systems. Editing at both day 10 and 14 on Dox was significantly higher when compared to the no Dox group (^*^p = 0.04, Kruskal–Wallis). Editing after 10 days on Dox with the gRNAi vector compared to the non-inducible vector (gRNA) was not significantly different between the two groups (*ns* = *p* = 0.51, Kruskal–Wallis). The same was found for the 14 day time point (*ns* = *p* = 0.28, Kruskal–Wallis).

Next, we wanted to assess if the gRNAi virus could be used *in vivo* to control genome editing in a Dox dependent manner. In these experiments we used a previously developed Cas9 AAV, where Cas9 expression is under the control of a neuronal specific truncated Mecp2 promoter (AAVP_Mecp2_-Cas9) (Swiech et al., 2015). We produced AAV2/DJ8-P_Mecp2_-Cas9 virus and the AAV2/DJ8-gRNAi_Tet2_ virus pseudotyped as DJ8 serotype. Specifically, AAV2/DJ8-P_Mecp2_-Cas9 and AAV2/DJ8-gRNAi_Tet2_ were co-infused bilaterally into the BLA, each at a titer of ~2.5E12 GC/mL (1 μl/side). For a control, AAV2/DJ8-P_Mecp2_-Cas9 and AAV2/DJ8-gRNAi_Empty_ were infused in a similar manner. Half of the animals in the Tet2 group and half of the animals in the Empty group were placed on a diet of 1 g/kg of Dox. Ten and fourteen days following surgery, the animals were sacrificed, and their BLA were microdissected. The genomic DNA was isolated from the microdissected BLA tissue and subjected to PCR/RFLP analysis to assess genome editing at the Tet2 locus (Figure 5A). Animals that received the AAV2/DJ8-P_Mecp2_-Cas9 and AAV2/DJ8-gRNAi_Empty_ viruses did not exhibit editing of the Tet2 locus as expected. Animals that received the AAV2/DJ8-P_Mecp2_-Cas9 and AAV2/DJ8-gRNAi_Tet2_ viruses did exhibit editing at the Tet2 locus, but this time editing only occurred in cases where the animals received Dox, indicating that genome editing could be regulated in a Dox dependent manner (similar results obtained in 3 independent samples per group; *p* = 0.04; Figures 5B,C). To compare our inducible system to a non-inducible system, we co-infused mice with an AAV designed to express a gRNA from a U6 promoter and AAV2/DJ8-P_Mecp2_-Cas9 at the same viral titers as the above experiments and followed the same timeline as the above experiments. RFLP analysis revealed similar levels of genome editing at day 10 and 14 for the conventional non-inducible genome editing system (gRNA), compared to our inducible genome editing system (gRNAi) *in vivo* (similar results obtained in 3 independent samples per group; *p* < 0.28; Figures 5D,E). In these *in vivo* experiments, we observed ~20% genome editing. This in part is due to the fact that our viral vectors are targeting neurons selectively, due to AAV's natural tropism for neurons *in vivo* (de Solis et al., 2015) and the fact that Cas9 expression is controlled from the Mecp2 promoter which restricts its expression to neurons (Swiech et al., 2015). Within the brain, glia cells make up at least 50% of the cells (Azevedo et al., 2009), so in this case we would at best only expect 50% of the cells within the microdissected brain tissue to exhibit editing and that is only if all the neurons within this microdissected tissue were transduced by both viruses. Therefore the ~20% level of editing *in vivo* we are obtaining essentially means we are observing ~40% of the neurons undergo genome editing, and this is similar between our inducible system and the previously developed non-inducible system.

**Figure 6:**
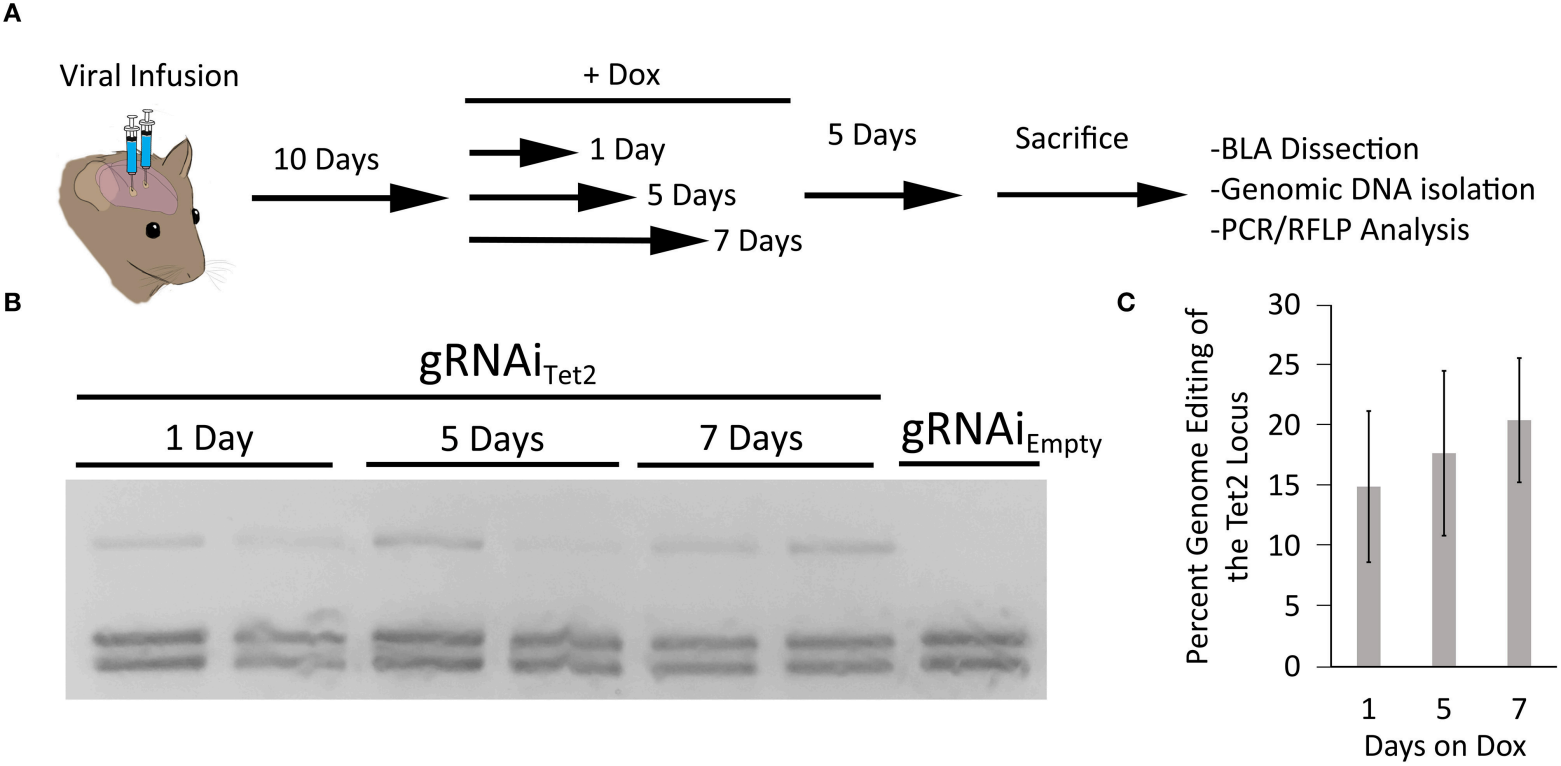
**(A)** Timeline from viral infusion to RFLP analysis of genome editing. Animals were infused into the BLA with AAV-P_mecp2_-Cas9 and AAV-gRNAi_Tet2_-TetR-GFP. Ten days post-infusion animals were placed on Dox food and then Dox food was removed either 1, 3, or 5 days later. Animals were sacrificed 5 days after removal of Dox and the BLA was microdissected and the genomic DNA was isolated and subjected to RFLP/ PCR analysis. **(B)** RFLP analysis of editing across 1, 5, and 7 days on Dox showed similar levels of editing and no editing in a gRNAi_Empty_ no Dox control. **(C)** Quantification of total edited DNA revealed similar editing across days animals were on Dox. These levels of genome editing were very similar to the levels seen during the 10 and 14 day editing time course for the gRNAi system and the non-inducible system. Percent genome editing was not significantly different between 1, 5, and 7 days on Dox (*p* = 0.67, Kruskal–Wallis).

In our last set of experiments, we wanted to determine the duration animals would need to receive Dox to induce genome editing. Therefore, in these experiments we infused AAV2/DJ8-P_*Mecp*2_-Cas9 and AAV2/DJ8-gRNAi_Tet2_ virus into the mouse BLA as described above. In this case, we waited 10 days following the infusion of the virus to administer Dox to the animals. The animals were then taken off Dox 1, 5, or 7 days later. The animals were sacrificed 5 days after Dox was removed from their diet, and the transduced cells within the BLA were microdissected. The genomic DNA was isolated from the tissue and subjected to PCR/RFLP analysis to assess genome editing at the Tet2 locus (Figure 6A). PCR/RFLP analysis (Figure 6B) revealed that day 1, 5, and 7 time points demonstrated roughly similar levels of editing (3 independent samples per group) (Figure 6C). These data indicate that this system requires as little as 1 day on Dox to induce editing. The degree of genome editing within this experiment was roughly comparable to the degree of genome editing obtained with the inducible and non-inducible gRNA viruses utilized in the experiments described in Figure 5E.

## Discussion

Here, we report the creation of an inducible CRISPR/Cas9 system that can be delivered to cells *in vitro* and *in vivo* via AAV. Genome editing in this system is regulated by a doxycycline inducible gRNA. We examined editing *in vivo* within the mouse brain at 10 and 14 days post viral infusion and observed a similar level of editing between both time points and the amount of editing was very similar and comparable to a non-inducible system. We also determined that this system is highly dependent on Dox to initiate genome editing *in vitro* and *in vivo* and therefore there does not appear to be any genome editing when Dox is not present. Administering animals Dox containing food for as little as 1 day (following 10 days post viral infusion) yielded roughly similar levels of genome editing to all other time points tested. We believe this system could be extremely useful for *in vivo* studies where regulating CRISPR/Cas9 genome editing in a temporally and spatially restricted manner would be beneficial. Certainly, behavioral neuroscience could benefit from this system because it would allow a gene to be knocked out at a specific time point during an ongoing behavioral assay that may span days to weeks.

Initially, we attempted to regulate the expression of Cas9 using a tetracycline response element (TRE) containing promoter; however, we determined that genome editing could not be regulated in a Dox dependent manner, due to the leakiness of Cas9 expression within this system. Some studies have described regulating Cas9 expression utilizing TRE containing promoters in other systems fairly successfully with limited leakiness (González et al., 2014; Dow et al., 2015). In these cases, the TRE—Cas9 transgene was stably integrated into the genome, which might reduce the observed leakiness because the transgene copy number may be lower. In comparison, ectopic expression methods such as plasmid transfection and viral transduction, can deliver hundreds to thousands of copies of plasmid or virus, and this may lead to an increase in leakiness, as seen in our experiments since the transgene copy number is so high. It is also possible that the low level Cas9 expression we observed in the non-induced state is driven in part by low level promoter activity from the inverted terminal repeat (ITR), since it's well established that the AAV ITRs can serve as weak promoters for RNA polymerase II (Wang et al., 1999).

Several inducible systems have been developed for CRISPR/Cas9 based systems where either the expression of Cas9 or the gRNA are inducible; however, none of these systems, to date, have been adapted for AAV delivery. Kiani et al. demonstrated that gRNA expression could be regulated from a polymerase II, TRE3G promoter in CRISPRi experiments conducted in HEK293 cells (Kiani et al., 2014). More systems have been developed where Cas9 expression or activity is regulated. One such system is light inducible, in which Cas9 is present in cells in an inactive or incomplete form, and with the addition of light, Cas9 becomes active and is able to complex with the gRNA and edit the target gene *in vitro* (Nihongaki et al., 2015). Qi and colleagues developed an anhydrotetracycline (aTc)-inducible Cas9 expression system for use in bacterial CRISPRi experiments (Qi et al., 2013). Davis and colleagues developed a system where inactive Cas9 becomes active in the presence of tamoxifen to enable genome editing (Davis et al., 2015) and Zetsche and colleagues developed an inducible system where Cas9 becomes active upon the addition of rapamycin (Zetsche et al., 2015). The tamoxifen inducible system in its current form would not be amenable to AAV delivery, due to AAV's genome packaging limits and the rapamycin inducible system would not be an ideal system to use *in vivo*, given rapamycin inhibits the mammalian target of rapamycin (mTOR), which is essential for many cellular processes (Ballou and Lin, 2008; Laplante and Sabatini, 2009) including those for memory formation (Nader et al., 2000).

To our knowledge, this is the first example of an inducible AAV mediated CRISPR/Cas9 system where the expression of the gRNA is regulated *in vivo*. One of the great features of this system is that the inducible gRNA vector (gRNAi) we developed might be cross compatible with many existing *S. pyogenes* Cas9 (SpCas9) systems. Coupling our gRNAi AAV vector with these systems may enable them to be inducible as well (i.e., Cas9 mouse, CRISPRi, etc.).

## Author contributions

CD and AH engineered the viral plasmids and purified the viruses, performed the ICC, and genome editing experiments. CD and RH performed the viral infusions into mice. CD and JP conceived the study and participated in its design and coordination and drafted the manuscript. All authors read, edited, and approved the final manuscript.

## Funding

This project was supported by NIH grant RMH100650A, RMH109945, the Texas Biomedical Device Center and the University of Texas at Dallas.

### Conflict of interest statement

The authors declare that the research was conducted in the absence of any commercial or financial relationships that could be construed as a potential conflict of interest.
